# Private Investments, Public Goods: Regulating Markets for Sustainable Development

**DOI:** 10.1007/s40804-021-00236-w

**Published:** 2022-02-28

**Authors:** Celine Tan

**Affiliations:** Reader in Law, School of Law, University of Warwick, Coventry, UK

**Keywords:** Aid, Environmental, social and governance, Financial markets, International development, Sustainable development goals, Public finance

## Abstract

In the new ecosystem for financing the sustainable development goals (SDGs), private actors are no longer passive bystanders in the development process, nor engaged merely as clients or contractors but as co-investors and co-producers in development projects and programmes. This ‘private turn’ in the financing of international development and other global public goods sees the enmeshment of public and private finance that brings aid and other forms of official development finance into sharp contact with regulatory regimes commonly associated with commercial investments, capital markets and corporate activity. The shift away from public resources for financing (e.g., multilateral sovereign loans) to leveraging financial markets for development capital (e.g., equity and portfolio investments) will insert countries into global financial markets and engagements with corporate actors in ways that will change forms of regulation, accountability and transparency of public finance. Zooming in on the creation of markets for sustainable development investments (SDI), this paper explores how this broader ‘reengineering of public finance’ is establishing new forms of governance that are restructuring the relationship between states and markets and between transnational capital and their host communities. Specifically, the movement towards private investments and financial markets as key drivers of financing for sustainable development has two critical impacts on transnational governance: (a) the use of private markets, in their capital allocation roles, as quasi-regulatory tools for achieving the SDGs and other global public goods; and (b) the deployment of private regulatory regimes (e.g., contracts, codes of conduct, corporate governance codes) as mechanisms to govern the social and environmental externalities of transnational economic activity. These developments have wide-ranging impacts on the domestic legal, political and civic constitution of states that can paradoxically constrain fiscal and policy space for enabling the attainment of the SDGs.

## Introduction

A significant shift is taking place in the landscape of international development finance. Premised on the notion of public–private partnerships, this latest evolution in global public policy sees the growing involvement of private actors in the mobilisation, disbursement and delivery of financing for sustainable development and other global public goods. In what has been referred to as the ‘new ecosystem of investment for sustainable development’,^1^ private actors are no longer passive bystanders in the development process, nor engaged merely as clients or contractors but as co-investors and co-producers in development projects and programmes.

This strategy of enabling private financing for sustainable development has intensified recently as the global community faces the critical task of pandemic response and recovery. There is an emerging consensus among major international development financiers that constraints on public resources in the foreseeable future mean that reliance cannot be placed on official sector flows to meet the enormous financial challenges faced by countries in the post-pandemic, climate critical world. Accordingly, mobilising private finance is seen as critical to responding to global social, economic and humanitarian needs, especially in developing countries, and tackling global collective action problems, including pandemics and the climate crisis. The private sector, especially private investors, is now viewed as a necessary partner in global efforts to ‘rebuild’ the global economy in the wake of COVID-19.^2^

An important component of this private turn in international development is the harnessing of national and international capital markets to mobilise investments in SDG and climate-related sectors through the promotion and development of sustainable development investments. Tapping into commercial debt and equity markets via sustainable and socially responsible investing and impact investing is seen as key to generate the leap from ‘billions to trillions’^3^ in the resources available to attain global public policy objectives, notably the sustainable development goals (SDGs) and climate change mitigation and adaptation.^4^

This paper focuses on the relationship between international development policy and practice and the creation of markets for sustainable development investing (SDI).^5^ It considers the active role played by official sector development actors – international financial institutions (IFIs), multilateral and bilateral development agencies and international organisations – in facilitating, incentivising and funding the shift towards financial markets as sources for SDG attainment. The paper explores how this broader ‘reengineering of public finance’^6^ is (1) establishing new forms of governance that are restructuring the relationship between states and markets and between transnational capital and the host communities for transitional capital; and (2) how these emerging governance formations impact on global distribution of resources for public policy goals.

Specifically, the paper argues that the movement towards private investments and financial markets as key drivers of financing for sustainable development has two critical impacts on transnational and domestic governance. First, the shift to financial markets as sources of capital for collective public goods and services is shifting the resource allocation role of states to private actors with attendant question marks over corresponding regulatory oversight of this quasi-public function. Second, the emergence of financial markets as primary sources for financing sustainable development is resulting in the deployment of private regulatory regimes to govern both social and environmental externalities of transnational economic activity as well as the sustainability of financial flows and funding for public goods and services.

The paper argues that these transformations have wide-ranging impacts on the domestic legal, political and civic constitution of states that can paradoxically constrain the fiscal and policy space available for enabling the attainment of the SDGs and other ecological sustainability goals. The movement towards financial markets as key sources for SDG financing is also likely to constrain the civic voice and participation of local communities in resource allocation and to undermine national ownership of social, economic and environmental policymaking while risking access to mechanisms for redress and accountability for harmful financial and operational acts of development projects and programmes.

The concern of this paper is therefore less about the endogenous development of SDI regimes and the general incorporation of sustainability and environmental, social and governance (ESG) considerations into investment decisions or about enacting behavioural change in financial market actors to align with collective global development and public goods targets. Instead, the focus is on the shift away from public finance and publicly allocated capital towards reliance on private financial markets to provide the resources to meet global public goods, and the impact this will have on meeting the SDGs and other sustainability objectives. The paper focuses specifically on the securities markets for sustainable development investing.

The paper proceeds as follows. The next Section provides an overview of the ‘private turn’ in international development finance and discusses how the SDG financing agenda is creating markets for public goods investments, locating this within the historical and contemporary role of international development policy and practice in restructuring regulatory and governance infrastructure in developing countries. Section 3 explores how these interventions in the modalities of global public goods financing are reorienting the role of the state and civil society in developing countries and discusses the implications for governance and public policy administration in these countries. Section 4 considers the regulatory gaps in the shift towards financial markets as providers of SDG resources and the effect of the enmeshment of public and private finance that brings aid and other forms of official development finance into sharp contact with regulatory regimes commonly associated with commercial investments, capital markets and corporate activity. The final section concludes.

## Private Finance for Sustainable Development

Although the ‘private turn’ in international development finance has longer historical roots,^7^ it has gained significant momentum with the inception of the UN 2030 Agenda for Sustainable Development (Agenda 2030) and the aforementioned SDGs and the Paris Agreement. The impetus to overcome the gaps between the ambitious SDGs and climate change mitigation and adaptation targets and the massive financial investments needed to meet them precipitated calls to mobilise financial capital beyond official sector finance. Private finance is seen as the key to scaling up resources to meet the challenges of these two major global agreements and the role of private finance is recognised and embedded within their implementation blueprints. For example, the Addis Ababa Action Agenda (AAAA), which sets out the framework for SDG implementation, underscores the importance of private finance in meeting long-term financing needs and commits states to developing regulatory and policy frameworks to enable private investments for sustainable development while encouraging the promotion of public–private partnerships in the financing of SDG needs, such as infrastructure and clean energy.^8^

### Public Incentives for Private Capital

A corollary of the evolving architecture of international development finance is the shifting role of official financing away from direct funding of development and other global public goods towards brokering financial investments in these areas.^9^ Operationally, this has meant the channelling of official finance, including concessional finance (also known as official development assistance or ODA), into (1) private investments and public–private partnerships (PPPs), particularly through bilateral or multilateral development finance institutions (DFIs);^10^ and (2) technical, policy and regulatory reforms or ‘upstream’ activities which create ‘enabling environments’ for private investments in development projects.^11^ The mobilisation of private finance has thus evolved into ‘a development policy goal in its own right’ with official financiers increasingly viewing private finance mobilisation as a key component of their *raison d’être* and a central part of their operational mandate.^12^

These developments have dovetailed with the exponential growth of the SDI market in recent years with green, social and sustainable bond issuances reaching a cumulative US$1.7 trillion at the end of 2020, doubling the amount issued in 2019 and expected to rise further in 2021.^13^ The resulting architecture has been termed a ‘framework for SDG aligned finance’ that is premised on the integration of ‘double materiality’ (financial and non-financial returns) in private finance decision-making and investments.^14^ In this emerging landscape, official development agencies and international organisations are being reoriented towards creating the enabling environment at global and national levels to tap into the estimated US30.7 trillion worth of assets under management^15^ available to finance sustainable development.^16^

This transformative change in the trajectory of international development policy and practice has been described as the movement from the Washington Consensus model of development to the Wall Street Consensus model of development. Where the Washington Consensus sought to ‘escort private capital’^17^ into developing countries through the restructuring of policy and regulatory regimes (for example, through deregulation and liberalisation policies of structural and sectoral adjustment conditionalities),^18^ the Wall Street Consensus is seeking to do so by providing financial incentives and regulatory interventions that aim to ‘de-risk’ private investments for public goods.^19^ As discussed in the following Section, this is often done at the granular level to foster ‘conditions of bankability’,^20^ creating new or adapting existing financial instruments to facilitate investor appetite in SDG investments while developing or restructuring the regulatory frameworks to constitute these new instruments and markets and to codify investor/creditor rights and mitigate or manage risk to private investments in this new SDG financing ecosystem.^21^

In this manner, the Wall Street Consensus extends the Washington Consensus policy prescriptions and interventions towards the creation of an enabling environment for financialised capital. It reflects the broader shifts in the global economy away from bank/relational-based modalities of debt financing towards market-based finance as a source of financing global economic activity. The SDG financing agenda must be located within the broader context of the ‘assemblage of private development finance’, the complex and interlocking framework of discourses, policies, programmes, facilities and practices that seek to rationalise, endorse and embed the prominent participation of private actors in the mobilisation, disbursement and delivery of development finance.^22^

As with the Washington Consensus, law and regulatory regimes are central to the constitution of this new landscape of financialised development, providing the ‘legal code’^23^ for creating and structuring assets for SDI investments and providing the regulatory framework for constituting and deepening SDI markets^24^ in order to ‘open up new circuits for financial investment, speculation and extraction’.^25^ The role of law and regulation in this context is to render social, economic and ecological sectors in the Global South accessible and investible to global capital through the apportionment, creation and enforcement of legal rights to assets and the establishment of a conducive domestic and transnational environment in which those conditions of bankability are promoted and protected.^26^

### Creating Markets for Public Goods Investments

Financial markets are central to the implementation of the emerging hybrid financing architecture for sustainable development. The movement away from official sector funding, such as grants and sovereign loans and guarantees, towards private commercial and non-profit financing, such as commercial loans, bonds and other securities, and ‘blended finance’ instruments and PPPs, has resulted in a greater reliance on debt and equity markets for resourcing SDGs and other global public goods. Financial markets are viewed as critical intermediaries between capital and SDG financing needs, serving as important sources of finance for development financiers *and* recipient states and communities. International policymakers have increasingly focused on scaling up access to and deployment of these sources of financing for SDG-related sectors.^27^

Correspondingly, since the acceleration of the ‘private turn’ in development finance, there has been significant investment of official sector resources – financial and technical – in the construction of sustainable finance markets with the aim of creating a large liquid and credible asset class for SDG investments.^28^ Specifically, in recent years, there has been a concerted emphasis on debt securities – corporate and sovereign bonds – as important vehicles for channelling capital to developing countries. International development financiers have engaged in the construction of SDI markets through: (1) participation in financial markets as issuers of SDI bonds; and (2) financial and regulatory interventions in global and domestic financial markets through their financing and technical assistance operations. The latter includes the development of policy and regulatory tools to create SDG-specific asset classes^29^ to incentivise SDI investments and enhance countries’ ESG ‘readiness’.^30^

First, IFIs, multilateral development banks (MDBs) and bilateral development agencies have started issuing SDG, green and sustainability-related bonds to finance their operations. While most MDBs and regional development banks have traditionally raised funds from international capital markets, their issuance of so-called ‘labelled bonds’ is relatively new and has accelerated with the development of the sustainable finance frameworks by the International Capital Markets Association (ICMA).^31^ The World Bank, for example, has been at the forefront of issuing green bonds since 2008 through its main lending facility, the International Bank for Reconstruction and Development (IBRD), and its private sector arm, the International Finance Corporation (IFC),^32^ but has recently issued sustainable development bonds (IBRD) that are consistent with the ICMA’s Sustainability Bond Guidelines (SBGs)^33^ and social bonds (IFC) that are aligned with the ICMA’s Social Bond Principles (SBPs).^34^ Regional development banks, such as the Asian Infrastructure Investment Bank (AIIB) and Development Bank of Latin America (CAF), have also similarly issued green, SDG and social bonds respectively.^35^

Additionally, in 2018, the World Bank’s concessional financing arm, the International Development Association (IDA), which has traditionally drawn its funds from donor replenishments and IBRD contributions, issued its first benchmark bond, marking its debut foray into the SDI capital market.^36^ Bilateral development agencies have also similarly tapped into sustainable finance markets to expand their capital base. France’s *Agence française de développement* (AFD), for example, issued a climate bonds framework in 2017 and an SDG bond framework in 2020 to raise capital for onward lending to sovereign and non-sovereign entities for eligible projects ringfenced by the terms of its climate or SDG bond ‘use of proceeds’.^37^

Aside from mobilising capital for their own operations, the participation of international development agencies in SDI markets sends important signals to other market participants. Leveraging existing operational and regulatory frameworks for SDG finance, SDG-linked securities issued by multilateral and bilateral development financiers lend credibility to emerging SDI markets and promote confidence in nascent normative frameworks linked to sustainable finance, notably the private regulatory regimes governing SDI markets, such as the ICMA sustainable bond and sustainable loan frameworks and third-party audit mechanisms (see further discussion in Sect. 3). In fact, DFIs, such as the IFC, have been central to the development of these sustainable finance standards, such as the Green Bond Principles (GBP), and are important standard-setters in the industry, building on their pioneering roles in developing sustainable lending frameworks, notably the Equator Principles, the environmental and social risk management framework for project finance.^38^

This market-building function of official development financiers is complemented by their operational policies and practices which have involved the deployment of funds towards the development of *financial* and *regulatory* incentives to mobilise private investments in green, sustainability and other SDG sectors. Driven by the overarching rationale that public finance, including concessional official development assistance (ODA),^39^ can act as financial and regulatory lever to ‘crowd in’ private sector finance for SDGs, policy and operational interventions by major development actors have focused on the use of *financial* and *regulatory* incentives to steer private capital into SDG sectors and to use private regulatory regimes to manage and supervise the engagement of private actors in what were previously state-dominated SDG sectors. This catalytic role of public finance is driven at both the ‘systemic, market-level’ and the transactional ‘deal-level’.^40^ Where the former aims to create enabling policy and institutional and market environments for private capital, such as through legal, regulatory and policy reforms, the latter involves the deployment of specific interventions to directly engage ‘private actors for individual investment projects’.

A key rationale for capital market interventions by development financiers is to reduce ‘investors’ informational and operational costs’ of participating in SDI markets^41^ and improve the risk profile of SDG investments to leverage private finance.^42^ As a recent study on green capital found, institutional investors from developed countries searching for green assets ‘want EM level yields without accepting EM level risk’.^43^ Policy, regulatory and financial interventions have therefore been used to: (1) create pipelines of ‘investible’ projects for SDI markets, including through the securitisation and pooling of SDG debt; utilisation of ‘risk-sharing’ instruments, such as joint issuances, guarantees and first-loss capital in labelled bonds; and provision of technical assistance and project preparation support; and (2) support the establishment of ‘mainstream SDG investment’ markets through, *inter alia*, the development of regulatory standards and audit mechanisms at national and international levels, including the development of ESG benchmarks and taxonomies for SDI bond issuances.^44^

Examples of IFI and MDB-backed capital market interventions include the issuance of a social bond by Ecuador in March 2020, backed by a partial credit guarantee from the Inter-American Bank (IDB), with proceeds to be allocated to addressing the country’s housing deficit,^45^ and a privately placed blue bond issued by the Seychelles to finance sustainable marine and fisheries projects that is partially guaranteed by the IBRD and supported by a concessional loan from the Global Environmental Facility (GEF) that covers part of the interest payments for the bond.^46^

The IFC has also actively supported the development of local capital markets through collaborative financing mechanisms, such as the Amundi Planet Emerging One (EGO) green bond fund that combines risk-adjusted green bond investments with capacity and market- building activities funded by the Green Bond Technical Assistance Programme (GB-TAP).^47^ The latter is a donor-funded initiative aimed at providing technical assistance and capacity-building initiatives to develop green bond markets, including developing ESG ‘indicators and tools for emerging market issuers of bonds and other products’.^48^ In Ghana, the GB-TAP is supporting the Securities and Exchange Commission (SEC) in the development of guidelines for issuers and investors of green bonds in Ghana.^49^

As discussed in Sect. 2.1, at the heart of these initiatives is the creation and establishment of markets for sustainable development and other public interest investments. This is leading to the transformation of official development agencies from *funders* of social and economic development and ecological sustainability to *brokers* of financing for these global public goods and services. IFIs, MDBs and other donors have increasingly conditioned public financing on ‘regulatory, policy and governance-related reforms in the context of private sector engagement’.^50^ This approach to development policy is encapsulated in the World Bank’s ‘Maximising Finance for Development’ or ‘Cascade’ approach which sees the Bank stepping in to provide public loans or guarantees for development projects only as a last resort after regulatory and policy reforms and the deployment of financial incentives have not leveraged the necessary commercial finance for these projects.^51^

Additionally, non-financial development agencies are also progressively reorienting their roles away from direct development operations towards facilitating private finance in SDG sectors. For, example in 2021, the UN Development Programme (UNDP), in collaboration with the UN-sponsored Global Investors for Sustainable Development (GISD) Alliance, launched the SDG Investor Platform as a means of providing private investors with country-level information to scale up sustainable development investments. UNDP country offices will be utilised to ‘lead research and preparation of market intelligence for private sector investors to translate country level SDG gaps and priorities into private sector investment opportunities’ and to convene ‘[p]ublic–private policy dialogues […] to identify recommendations to improve the enabling environment for SDG aligned investments’.^52^

## Governing Markets for Sustainable Development

The reliance of low and middle-income countries on external finance renders them vulnerable to shifts in global economic conditions but also to changes in the international law and policy governing the global economy, including law and policy relating to the distribution and allocation of global capital and financial resources. International development policy, practice and governance have long been influential in shaping the economic development trajectories and social and political organisations of developing countries in the Global South,^53^ with attendant law reform and regulatory interventions that accompany countries’ receipt of external finance and participation in cross-border economic activity acting as significant drivers in reconstituting domestic regulatory regimes and countries’ engagement with the exterior.^54^

Cumulatively, the reorientation towards private financing for sustainable development represents a progressive privatisation of international development finance that can lead to what Karwowski has called the ‘financialization of the state’.^55^ Defined broadly, the process of privatisation involves the transfer of assets or functions, in whole or in part, from the public or official sector to the private or non-state sector, involving ‘the increased reliance on private actors and market forces to pursue social goals’.^56^ Meanwhile, state financialisation can be described as ‘the increasing influence of financial logics, instruments, markets and accumulation strategies in state activities’, including in the design of fiscal and monetary policies.^57^ These twin processes of privatisation and financialisation can reconstitute, and have reconstituted, the modalities and governance at the market, state and transnational levels, with significant implications for public accountability and scrutiny and for the legal and regulatory oversight of government and private entities involved in the mobilisation, disbursement and delivery of sustainable development finance.

### Displaced Engagement and Ownership

First, the movement away from public resources for financing to leveraging financial markets for development finance is changing the role and function of the state in developing countries, particularly in its revenue-generation and resource-distribution roles. There are concerns that many under-resourced and capacity-constrained developing countries will struggle with the legal and regulatory transition to these new private modalities of finance with adequate public interest safeguards.

Moreover, as financial markets constitute an ever-increasing share of resources available for essential services, social provision and environmental sustainability, they will also increasingly serve as quasi-regulatory tools for access to and use of such global public goods, structuring the terms and conditions on which resources are allocated and utilised. These terms, as Davis has observed in relation to development finance more generally, are important in determining the impact of development finance as they ‘define the balance that has been struck between the potentially conflicting interests of the various actors implicated in the transaction, namely, financiers, intermediaries, ultimate recipients of funds and other residents of developing countries’.^58^

Scholars of privatisation and financialisation of public services have long cautioned that shifting the responsibilities from the government to the market for the financing and provision of public goods and services fundamentally alters the process and outcomes of distributive contests within states and communities as well as the underlying regulatory and administrative architecture governing fiscal and monetary policy and the provision of domestic and global public goods and services. As Karwowski argues, ‘how public revenue is raised directly impacts on how public expenditure is shaped and welfare policies designed’, resulting in a ‘democratic deficit’ that undermines oversight and scrutiny over decisions taken in relation to the mobilisation and deployment of public finance and relatedly, in the context of financial markets, potential contracting of sovereign debt or other contingent liabilities on the state.^59^

The turn to financial markets for sustainable development finance can and does displace national ownership of and participation in social and economic policymaking, including SDG sectors. Decisions on which sectors to prioritise in fiscal decision-making will increasingly be determined not by public institutions or elected representatives or communities but by institutional investors, asset managers and, increasingly, index providers, credit rating agencies, and financial actors and private investors based in advanced economies. As scholars and observers have noted, debt-based financing for sustainability and social provision is a double-edged sword. While it can offer financial, legal and regulatory tools to influence corporate and sovereign conduct vis-à-vis meeting SDG and other global public objectives, it can also constrain the exercise of state authority over external finance and limit public and community engagement in the prioritisation of public finance and design of social, economic and environmental policies and programmes.^60^ These new modalities of finance not only ‘transfer resources from public to private investors’, they also ‘remove social provision from democratic decisions and scrutiny into the realm of financial markets’.^61^

The United Nations Conference on Trade and Development (UNCTAD) has warned that the SDG financing agenda as oriented towards private investments can subordinate countries’ ‘ownership of development policy’ to the interests and priorities of private actors, particularly those based in OECD countries that have been heavily influential in reshaping the aid architecture and prioritising private finance for development.^62^ It argues that the more SDG investments are channelled through private actors, the more these investment projects and programmes are ‘disconnected from country development plans’ as DFIs, the main intermediaries between private finance and SDG investments on the ground, often bypass state agencies to contract directly with the private sector in the host state.^63^

At the same time, the segmentation and earmarking of income derived through SDI arising from the contractual provisions of labelled bonds, notably the use of proceeds and project evaluation, selection requirements and attendant reporting criteria,^64^ can place constraints on governments’ ability to manage public finance as well as to respond to domestic financing needs and meet community demands for investment in specific SDG sectors that may not necessarily fall into earmarked funds from labelled bond issuances. In addition to donor-driven policy and regulatory reforms, private governance regimes regulating sustainable finance markets can also exercise significant leverage over developing country governments and can undermine rather than complement or strengthen domestic SDG-related policymaking.

Most of the policy and regulatory guidance for the establishment of SDI markets has emerged from public and private commercial or non-profit entities based in OECD and other major capital-exporting states. Labelled bond markets and the market for impact investments are governed primarily by ‘a constellation of quasi-regulatory standards, procedures, and institutions’ that are loosely based on private corporate social responsibility (CSR) regimes and ESG frameworks developed by public and private entities in advanced market economies.^65^ This can lead to what Soederberg has termed the ‘new conditionality’ or the ways in which market indices and non-financial benchmarking that are used to guide socially responsible investing can reproduce or exacerbate the dynamics of power inherent in traditional donor-recipient relationships.^66^

The rapidly growing interest in sustainable and socially responsible investing has led to a proliferation of ESG data and service providers that remain, for the most part, dominated by private providers or industry associations and unregulated by the public sphere.^67^ The benchmarks, indicators and taxonomies used to develop SDG sectors into asset classes for sustainable development investments (see Sect. 2.2) act not only as ‘market-building’ devices^68^ but also as market-screening or gatekeeping tools that can paradoxically exclude and constrain access of countries and communities to external capital.^69^

Indicators and benchmarks can therefore act as technologies of governance and regulation^70^ in the SDI market, changing decision-making processes ‘by altering the forms of knowledge that are relied upon by decision-makers’.^71^ Indicators can serve as ‘norm-vessels that transport social and environmental “value” into commercial investing’ and ‘creating new forms of knowledge’ to inform investors’ decision-making about what is an ‘investible’ social or environmental product.^72^ Accordingly, private bodies and industry associations active in the construction of SDI markets, such as the ICMA, the Sustainable Accounting Standards Board (SASB), Impact Reporting and Investment Standards (IRIS), and the Global Impact Investing Rating System (GIIRS), can and do exert enormous influence over where capital is deployed and into which sectors in developing countries.

Private regulatory authority in the SDI market is also exercised by financial indices that track the performance of green, social and sustainability bonds, a form of ‘informational regulation’ that relies on corporate and sovereign issuers responding to signals produced by the aforementioned ESG rankings or ratings and adapting corporate or sovereign behaviour accordingly.^73^ Like ESG data providers, there has also been a proliferation of ESG-titled indices, including from major index providers such as MSCI, S&P and FTSE Russell.^74^ Indices as gatekeepers to capital ‘are reputational intermediaries’ between issuers and investors and their ‘prescriptive authority’ is based on the threat of removal of non-compliant securities from an index.^75^ These indices have become increasingly influential in global capital allocation in the movement from active to passive funds as decision-making about where to invest in the latter is delegated to indices that can ‘de facto steer capital’ through inclusion or exclusion of firms and countries that can cause large quasi-automatic capital inflows or outflows from countries.^76^

The displacement of government decision-making and oversight in public revenue raising and public expenditure management resulting from the shift from official to private sector financing, including the diversion of ODA and other official development finance towards leveraging private finance instead of direct funding of SDG needs, is contrary to long-standing commitments underpinning international development cooperation. National ownership of and inclusive participation in social and economic development policymaking have been key principles underpinning international development cooperation and enshrined in numerous agreements, including the Paris Declaration on Aid Effectiveness, the Accra Agenda for Action and the Addis Ababa Action Agenda.^77^ It is difficult to see how a dispersed framework for financing sustainable development that is primarily dominated by the interests of OECD and other major ‘donor’ states and operationalised chiefly through private governance regimes outside the oversight of financier and recipient governments meets the development cooperation commitments established by the international community.

### Outsourced Oversight and Accountability

**Fig. 1:**
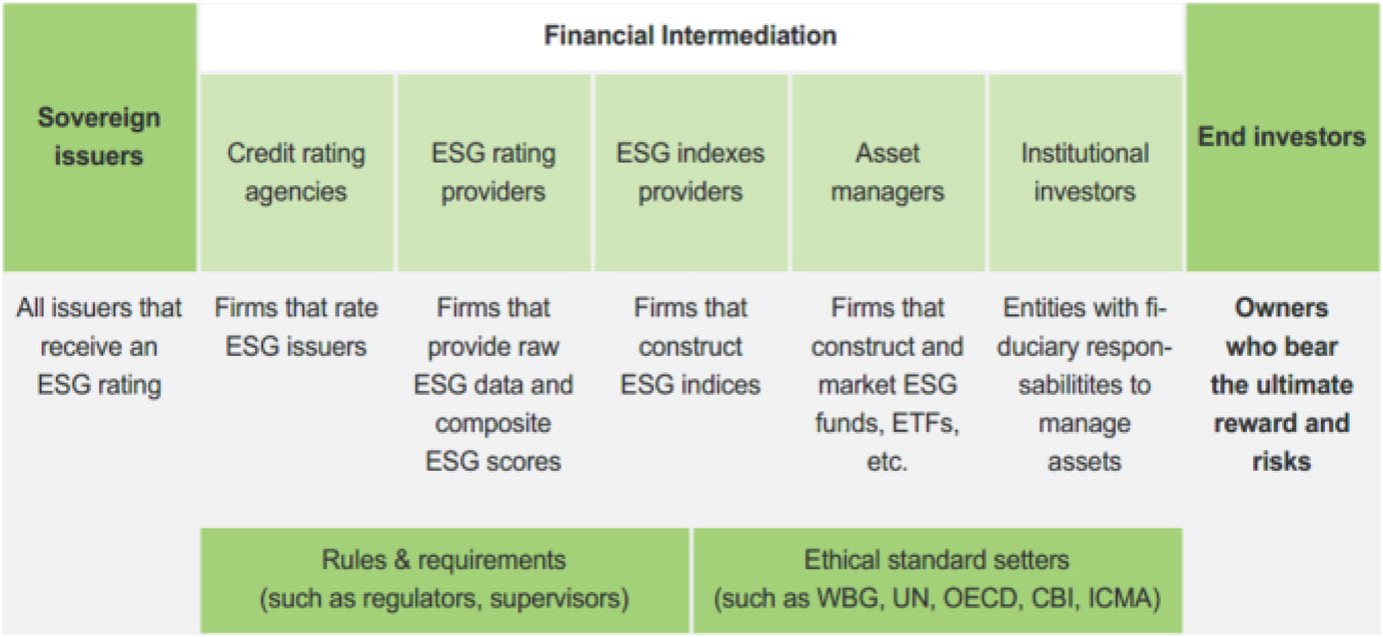
The structure of the ESG financial ecosphere. Source: Boitreaud et al. (2020)

A key feature in the shift towards private finance in sustainable development is the proliferation of private commercial and non-profit actors in the arenas where international development cooperation is negotiated, implemented and regulated. Private entities, including investors, corporate firms, non-profit organisations and industry associations, are becoming central to this hybrid development finance landscape, playing critical roles as financiers, service providers, regulators and decision-makers. This rapid diversification of actors in the sphere of development finance (see Fig. 1 for an example) and the fragmentation of financial sources, facilities and institutions are complicating domestic and international oversight and accountability of development finance and raises significant questions about the capacity of current legal and regulatory frameworks to govern this new regime and new non-state actors in development finance policy and practice.

The privatisation and financialisation of development finance exacerbate the already highly fragmented regulatory architecture of international development cooperation that is characterised by a diversity rather than unity of substantive and procedural rules and procedures and overlapping jurisdiction, parallel competences and differing levels of enforceability of rights and obligations.^78^ This not only creates significant operational burdens in recipient countries, particularly countries with weak administrative capacities (see Sect. 3.1), but also disperses attempts at securing accountability for the practice and outcomes of development finance, including monitoring of decision-making processes, transparency over financial disbursements and procurement of goods and services, and oversight over the implementation of development policies and projects.

Several concerns arise here vis-à-vis accountability of SDG finance delivered through financial markets. First, this reorientation has not been matched by robust measures to map, track and account for financial flows to countries in support of SDG and other global public goods. While there remain weaknesses in the current system of aid accounting, civil society pressure over the past few decades has ensured that there is now greater transparency of official resource flows to developing countries and the terms and conditions accompanying such flows with some measure of harmonisation across different official financiers, especially IFIs and MDBs. Major MDBs, such as the World Bank and regional development banks, have access to information policies that make most information on projects and programmes funded through loans and grants publicly available.^79^ OECD member states also provide relatively robust annual reporting of ODA and other official flows (OOF) to the OECD Development Assistance Committee (DAC).

In many cases, disclosure of information by external development financiers constitutes an important part of domestic accountability in developing countries, enabling access to financial and economic policymaking that will not otherwise have been made available through domestic channels. Thus, there is a concern that the dispersal of financing will disperse information about financial flows and dilute access to information and therefore oversight of executive fiscal decision-making within countries due to a combination of weak capacity and, occasionally, deliberate obfuscation. In recent years, there have been concerns about the transparency of private sovereign debt contracts and the impact of so-called ‘hidden debts’ on the debt sustainability of countries, particularly low-income and highly indebted states. For example, Mozambique remains embroiled in a series of litigation over previously undisclosed US$2 billion worth of private bank loans and bonds contracted by state-owned companies without the approval of the Mozambique parliament and backed by hidden government guarantees.^80^

Transparency concerns also arise because private sector investments, even when routed through official development finance institutions such as DFIs and public–private facilities or multi-stakeholder partnerships (MSPs), are subjected to weaker transparency and information disclosure regimes than their counterparts.^81^ Most DFIs, which on-lend proceeds from SDI bond issuances or injection of capital from governments to the private sector, are not subjected to the same standards of public disclosures and are ‘not obliged to share information with local authorities’.^82^ The IFC’s Access to Information Policy, for example, excludes the release of ‘commercially sensitive and confidential information’ which includes ‘financial, business, proprietary or other non-public information about its clients, its member countries or third parties’.^83^ The IFC argues that as a financial service provider to the private sector, it will be ‘contrary to the legitimate expectations’ of their private sector clients to disclose such information for ‘fear of compromising their projects or other proprietary information in a highly competitive marketplace’.^84^

Lack of disclosure also makes it difficult for communities affected by development projects and programmes financed by DFIs and their intermediaries to obtain accurate information about ESG risks and options for recourse to mitigate or remedy harms arising from these projects. A study by development finance watchdog Publish What You Fund found that while most DFIs have broadly transparent and well-developed ESG disclosure policies, there were significant shortcomings in policy implementation, particularly in the disclosure of ESG risks to affected communities.^85^ In other words, even where DFIs have relatively good disclosure policies at the global level (for example, through websites and institutional mechanisms), there is limited evidence that this information reaches project-affected communities, which can hamper access to local accountability.^86^

Current operational practice of DFIs demonstrates the problems with the use of ‘[d]isclosure-based mechanisms’, such as the aforementioned ‘reporting frameworks, labeling, and ranking/awards’ as a means of ‘informational regulation’ of SDI markets.^87^ Aside from the lack of standardisation in ESG frameworks, leading to what has been called the ‘“alphabet soup” of acronym-heavy measurement and reporting standards’,^88^ there is a question mark over the efficacy of such standards and codes in preventing ‘greenwashing’ or ‘SDG-washing’ in financial market lending as investors have little legal recourse for non-compliance with use of proceeds or management of proceeds provisions. Unlike sustainability-linked loans from banks, there is no contractual provision to penalise issuers for failure to comply with green or social obligations. For example, the provision to apply green bond proceeds to green projects ‘is not generally a contractual obligation’ unlike green loan obligations, so investors of green bonds have limited options if green bond issuances do not meet declared investment objectives.^89^

There are also deeper systemic problems with the current disclosure regime. SDI markets are currently primarily regulated by industry-led private governance regimes, which significantly increases the risk of regulatory capture by private interest groups.^90^ Stakeholders, including governments and communities, remain ‘passive beneficiaries’ of the standards and audit regimes established by investors and other market actors.^91^ Specifically, the labelling of public and collective social and sustainability services as ‘market opportunities’ leads to a ‘boundary shift’ that ‘profoundly alters the character of a service’ and transforms communities and the environment into commodities.^92^ Thus, accompanying the ‘inevitably complex contract arrangements’ of these new innovative financialised schemes for sustainable development is the transformation of policy accountability frameworks, ‘with governance and reporting systems geared toward the needs of private funders rather than elected officials’.^93^

For many developing countries, this form of privatisation and exercise of private authority over public policymaking and service delivery exacerbates the loss of policy autonomy discussed in Sect. 3.1. For individuals and communities, this shift ‘potentially alters the institutional framework through which citizens normally articulate, mediate, and promote their individual and shared interests’, with the impact of this ‘institutional restructuring’ falling differentially across different constituencies.^94^ In these countries, the provision of services is often the main process in which citizens and residents encounter the state, and the removal of the state from the financing and delivery of public services can have detrimental effects on the democratic and participatory policymaking that also constitute important commitments under the UN SDGs, AAA and other international agreements on development cooperation.

The primary focus of the private regime governing SDI markets has been on disclosure, reporting, certification and ratings and rankings, mainly to meet the financial and regulatory due diligence requirements of investors. What has been overlooked is the responsibility of investors for harms caused by projects and programmes funded by their investments. A ‘remedy gap’^95^ already exists within the traditional financial services sector where banks and other financial institutions may sit one step removed from project operations that may lead to displacement, social or environmental harms to local communities. This gap has been increasingly closing over the years, driven mainly by MDBs and DFIs promoting and operationalising ex ante ESG assessments as well as ex post accountability processes, including the promotion and establishment of institutional and project-level grievance mechanisms and independent accountability mechanisms (IAMs).^96^

While not without their limitations, the accountability mechanisms of MDBs and DFIs have become standard bearers in the financial industry, providing an important normative and procedural framework governing the financing of development projects and programmes and enabling a regime through which grievances against development actors can be evaluated and investigated and some form of redress can be obtained.^97^ Once again, here, the World Bank Group has been at the forefront of developing environmental and social safeguards and establishing mechanisms, such as the Grievance Redress Service (GRS) and Inspection Panel at the IBRD and IDA and the Compliance Advisor Ombudsman (CAO) at the IFC,^98^ which have been adopted by other MDBs and DFIs. However, the movement away from bank-based finance towards diversified and dispersed SDI market-based finance extends the chain of responsibility for harms, and fragments potential avenues of remedy for communities harmed by a project or programme ostensibly financed through a green, social and sustainability bond.

SDI securities mostly mimic ‘plain vanilla’ securities (see Sect. 4.1) and there is no recourse to the investor if individuals or communities are harmed in a project or programme funded through such means. Additionally, while external ‘impact’ measurement and third-party assurance providers can capture some negative externalities in their review of an SDI bond issuance framework which can lead to loss of investor confidence or interest in the bond, there is little redress for communities that have been harmed to seek mitigation or remedies for social and environmental harm and little recourse to the investors despite such securities being designated specifically as sustainable development investments. Accordingly, aside from screening or divesture, SDI bondholders have limited leverage over operational ESG practices of funded entities.

Meanwhile, the overwhelming focus of development agencies on SDI market-building and de-risking private capital has paradoxically marginalised concerns about ESG risks of funded projects to communities. This regulatory architecture addresses less the ‘risk-to-people’ than it does the ‘risk-to-corporation’^99^ while decentring communities from the processes of social investment and economic transformations that are purportedly aimed at improving their lives. The expectation that ESG risks in securities markets will be mitigated and addressed through traditional CSR practices, such as social audit regimes, has not been borne out by past experience. Studies of CSR audit regimes have found that while such frameworks may lead to incremental advances in addressing CSR concerns, such as labour, health and safety standards, in supply chains, the audit regime is designed more to minimise reputational risks to investors and enhance corporate legitimacy than to intervene in or resolve harmful practices.^100^ Further, there are concerns that audit regimes can actually harm communities by failing to carry out accurate and responsible audits, leading to the continuation or exacerbation of social (and environmental) externalities of corporate behaviour.^101^

## Regulatory Gaps and Market Failures

The shift to financial markets as an alternative to official sector funding for sustainable development must be located within a broader framework of the international financial architecture and the legal and regulatory framework which governs it. The privatisation and financialisation of development finance deepen countries’ exposure to international capital markets, increasing their porousness to shifting global financial conditions and the policy and regulatory responses to such developments by systematically important countries and international financial actors and networks. This has the potential of generating financial and regulatory risks not only for domestic financial sectors but also for the international financial system more generally, and can impede the resolution of financial and sovereign debt crises if and when they occur.

### Transmission Nodes for Financial Instability

The development and use of novel financial instruments to create and develop SDI markets can create new transmission channels for financial instability that can have adverse consequences both for the sustainability of individual financial interventions and for the broader financial and economic health of states in receipt of such financing. Despite the role played by the unregulated growth of innovative financial engineering, including derivatives and complex securitisation structures, in the build-up to the global financial crisis of 2007–08,^102^ there remains an under-appreciation of the financial risks posed by reliance on and proliferation of such financial instruments. This is especially pronounced when accompanied by the liberalisation and deregulation of domestic financial sectors that has been either a feature of structural and sectoral conditionalities attached to development finance or a result of commitments under the World Trade Organisation’s (WTO) General Agreement on Trade in Services (GATS) or under bilateral or regional free trade agreements (FTAs) or investment treaties.^103^

Developing countries are particularly vulnerable to the volatility in international financial markets due their relatively weak structural positions in the global economy as well as their lack of control over the fiscal and monetary policies in advanced economies and the marginalisation from the sites of decision-making in the international financial architecture.^104^ Even before the onset of the global COVID-19 pandemic, developing counties, especially emerging markets, witnessed heightened instability in cross-border capital transactions concomitant with their increased integration into global financial markets. Portfolio flows, cross-border bank loans and other debt instruments were and have remained particularly volatile for developing countries due their ease of cross-border entry and exit facilitated by the aforementioned liberalisation of capital account and foreign investment regimes in developing countries.^105^

The vulnerability of developing countries to the volatility of capital flows was demonstrated by the massive and sudden outflows of capital from developing and emerging economies (DEEs) at the start of the global pandemic in the first half of 2020 when almost US$103 billion was withdrawn from DEEs between mid-January and mid-May 2020.^106^ At the same time, the costs of servicing foreign-currency denominated debt have increased significantly as the cost of borrowing has soared for DEEs and investors look to shelter from the crisis in the ‘safe asset’ markets and higher yields of advanced economies.^107^ Developing countries’ experience with the behaviour of financial markets during the COVID-19 crisis mirrors past experience with financial crises and exemplifies the hierarchical and asymmetrical nature of the international financial architecture and the procyclicality of financial markets.^108^

Additionally, in efforts to create an enabling environment for private investments in developing countries, official sector financiers can and do accelerate their loss of policy and regulatory autonomy. The development of novel financial innovations, such as securitisation, which are increasing the availability of credit by ‘converting non-tradeable financial assets into tradeable securities, transforming liability risks into financial instruments and diversifying individual creditor risks’ to create SDI markets can lead to short-term speculation and domination of foreign financial actors in domestic markets that render countries ‘more vulnerable to the vagaries of international financial markets’ and subservient to private financial entities than ever before.^109^ This is leading to ‘a profound loss of control by developing country governments over the pace and direction of credit creation in their own economies’ and accentuating their exposure to volatile and unpredictable short-term capital.^110^

There is no evidence that the private ordering regimes governing the SDI market can or will overcome these structural deficits within the international financial system. Instead, the drive towards greater integration of developing countries into international capital markets, in terms of both contracting foreign currency debt by sovereign and corporate labelled bond issuances in external financial markets and the liberalisation of domestic capital markets to foreign investors, will continue to render countries vulnerable to global financial instability. Consequently, there are significant concerns that the exponential rise in the interest in and issuance of labelled bonds and other SDI securities is leading to an ‘ESG bubble’^111^ that can have significant financial and regulatory ramifications as well as harmful consequences for communities reliant on these instruments to fund social, economic and environmental goods and services.

The regulatory design of SDI markets which mimics conventional capital market regulation, discussed in Sect. 3.2, is unlikely to insulate developing countries from this vulnerability as these frameworks are designed to protect the interests of investors rather than those of the host states of SDI markets and ‘investment-affected’ communities, considered as ‘distant secondary beneficiaries’.^112^ Regulation of SDI markets is ‘primarily shaped by the very same market participants that sell, buy, trade or assess these financial instruments’,^113^ which has consequently resulted in the phenomenon known as ‘blueprinting’ whereby new markets – such as SDI markets – are ‘created based on a template that is set by an already-existing market, such as conventional finance’.^114^ While this ‘systemic mimicry’ is often understood as a deliberate exercise to incentivise investment ‘through the strategic use of language, institutions, and metrics that are familiar to investors’, there is a danger in converting tools made to measure and market conventional finance and apply them to social and sustainable finance.^115^

Aside from the potential for greenwashing and SDG-washing discussed in Sect. 3.2, ‘blueprinting’ conventional securities regulation for the SDI market can transfer the same regulatory deficits of these regimes to the SDI market, including oversight of third-party ESG assurance providers or labelled bond verifiers that are playing key roles in the allocation of ESG capital. Concerns have been raised about the governance and supervision of such ESG service providers that mirror the operations of credit rating agencies (CRAs), the failings of which have been widely attributed as contributing to the global financial crisis in 2007–08.^116^ Like CRAs, ESG data providers exert enormous influence over financial markets as their ratings provide investors with qualitative information to guide investment decisions, but unlike CRAs, ESG data providers are not publicly regulated.^117^ This lack of regulatory oversight can lead to poor quality certifications and inflated ratings of the kind that contributed towards the previous financial crises.^118^

Moreover, while SDG-labelled securities, such as green, social or sustainability bonds, are targeted at financing specific SDG or ESG sovereign or corporate expenditure, there is no provision which prevents investors from divesture due to financial considerations, even if such divestiture, particularly for large institutional investors, would mean ramifications for the beneficiary communities. Conversely, due to the regulatory expectations of asset managers and institutional investors and the role of credit ratings and financial indices in downgrading the investment ratings of sovereign and corporate debt in DEEs during times of crisis, divesture is often a measure that is imposed on investors by the regulatory authorities of their home jurisdiction. This short-term focus of financial market investors and the absence of contractual or regulatory mechanisms to mitigate these structural deficits and the cyclical nature of portfolio flows in the SDI markets are why the rush to replace direct, official sector financing of sustainable development with private capital may result in financial contagion that can jeopardise the attainment of SDGs themselves.

### Private Finance, Public Debt

Reliance on financial markets as a source of sustainable development finance can lead, and has led, to an accumulation of unsustainable debt in developing countries, exacerbating the systemic vulnerabilities of developing countries in the global economy. While having the potential to scale up resources, experience has demonstrated that private finance also has the potential for ratcheting up the external debt burdens of developing countries and complicating arrangements for sovereign debt crisis management and resolution. Private sector debt held by developing countries as a share of total sovereign debt had already almost doubled between 2000 and 2019 with corresponding rising debt servicing costs,^119^ and the turn to SDI markets will only contribute to this increased risk profile.

First, bond finance has been considered the ‘most volatile component’ of public debt due to the ‘strong speculative features of international financial markets’ as well as the terms of such financing which are more onerous than official sector finance with shorter maturities, higher and more variable interest rates and easier exit terms.^120^ As labelled SDI bonds follow structures and investment trajectories similar to those of traditional securities, they are likely to follow a similar profile, enabling such bonds to be traded with ease on primary and secondary markets.

Second, while the turn to private finance can shift debt off government balance sheets and the creation of domestic financial markets can reduce countries’ exposure to foreign currency-denominated debt, high levels of private debt can be of significant concern as ‘they represent a large contingent liability on public sector finance’.^121^ These contingent liabilities can be *express*, such as government guarantees, or *implicit* when widespread private sector indebtedness in a financial crisis is socialised or converted into sovereign liabilities as occurred in previous sovereign debt crises.^122^ This means that the financial and regulatory incentives discussed in Sect. 3.2 that are promoted by international development agencies as levers to catalyse SDG resources from the private sector may in turn generate additional liabilities and risk of debt build-up for developing countries in the longer term.

Third, the diversification of the creditor base will compound the international process for intervening in and resolving financial and sovereign debt crises that currently relies on the loose coordination of informal and voluntary negotiations between the sovereign debtor and its creditors.^123^ In the absence of a formal sovereign insolvency process, the introduction of new creditors and new debt instruments into a sovereign debt landscape is likely to complicate efforts to restructure sovereign debt. Unlike official sector debt, which is easier to restructure through channels such as the Paris Club, the introduction of a broad base of private creditors can lead to more disorderly debt restructuring in the event of a sovereign debt crisis, prolonging debt distress in affected countries and undermining social provision and jeopardising climate transitions.^124^ Thus, transitioning developing countries from official financing to commercial markets can impose significantly new and more onerous legal obligations on already struggling governments, as noted in Sect. 3.1.

Negotiations with private creditors, particularly bondholders, in the event of a sovereign debt crisis have historically been challenging and fraught given the absence of a standardised procedure to deal with such debt. As discussed, aside from provisions relating to the use of proceeds and meeting key performance indicators and monitoring requirements in market guidance such as the GBP or SBP, there are no specific contractual provisions that distinguish labelled bonds from ordinary sovereign or corporate bonds. SDI securities will therefore be subjected to the same treatment as other securities in a solvency crisis. Most notably, despite the importance of the sectors they purport to finance, including essential services such as health, education, water and energy infrastructure, there are no provisions to ensure that investors must enter into negotiations to restructure such debt in situations of unsustainable debt burdens in host states.

Once again, the COVID-19 pandemic has starkly demonstrated the perils of the shift from official financing to commercial financing for many low and middle-income countries. International organisations have warned of an impending global sovereign debt crisis as rising financing costs and erratic growth threaten already fragile debt positions of many DEEs, with many emerging economies servicing considerably higher interest rates than advanced economies, despite less borrowing on international financial markets.^125^ Many countries have also headed off immediate debt crises by borrowing heavily from official financiers, notably the International Monetary Fund (IMF), and accessed concessional debt relief facilities, such as the IMF’s Catastrophe Containment Relief Fund (CCRT) which writes off eligible IMF debt service for eligible countries.^126^

However, private creditors have so far failed to participate in multilaterally agreed debt relief measures and continued to profit from debt repayments, including from highly indebted states that are spending more in debt service repayments than on pandemic intervention and mitigation.^127^ At time of writing, none of the private creditors of the 73 eligible countries have participated in the G20 Debt Service Suspension Initiative (DSSI) – which provides for a temporary moratorium on eligible debt service payments – or the G20 Common Framework for Debt Treatments – which provides for restructuring of eligible debt.^128^ Many countries have also been reluctant to seek private sector debt relief due to the impact this would have on their credit ratings and subsequently on their future capacity to borrow on international capital markets, once again raising concerns about the operations of CRAs and other private regimes governing international financial markets.^129^

In response to the lack of private sector participation in pandemic debt relief measures, the Institute for International Finance (IIF) has reiterated calls for ‘improving the sovereign debt restructuring process’ through, *inter alia*, greater ‘public–private sector dialogue’ and more ‘debt transparency’ as well as the promotion of industry codes of conduct such as the G20 and OECD-endorsed Principles for Stable Capital Flows and Fair Debt Restructuring.^130^ Importantly, despite not addressing concerns raised about the contribution of private finance to debt sustainability in developing countries, the IIF emphasises the salience of ESG investing as a source of ‘sustainable capital flows’ to these countries.^131^ It highlights current efforts to ‘build a blueprint for scaling global sustainable capital markets across asset classes’, including labelled bonds, ‘money market products, derivatives and insurance solutions’ as well as ‘SDFG-linked and sustainability-linked instruments’ such as the novel ‘nature performance bonds’ that build on the so-called ‘debt-for-nature’ swap instruments and link sovereign debt relief with commitments to protect biodiversity in DEEs.^132^

While these new initiatives hold out promises to scale up financing for sustainable finance capital, like the other capital market instruments discussed in this paper, the broader concerns about the short and long-term impacts of these instruments within the broader framework of the global financial architecture and massive regulatory gaps which still exist will exacerbate rather than alleviate the financial and debt burdens of DEEs. The IIF proposals continue to rely on private governance regimes and the development of the aforementioned informational regulatory tools to supervise the operations of the new actors and new markets for sustainable development investing while placing considerable responsibility for managing debt on the debtor state and official sector agencies.

## Conclusion

Financial markets are being heralded as the panacea to the challenges faced by developing countries in securing resources for meeting the SDGs and other global public goods and sustainability targets. This turn to private finance in international development policy and practice has occurred in tandem with the increasing mainstreaming of ESG investing as financial markets are subjected to growing investor demand, policy and regulatory pressures, particularly in advanced economies, to pivot capital towards socially responsible and sustainable investments. Official sector financing has been routed away from direct funding of development projects and programmes towards brokering private capital for meeting sustainable development expenditure.

However, the development of SDI markets has not been matched by a corresponding development of regulatory mechanisms at domestic and international levels to mitigate the consequences of market failures in these sectors. The rapid integration of developing countries into global financial markets via SDI markets and international development policies encouraging them does not account for the specific vulnerabilities of these countries within the international financial system that remain unresolved despite several cycles of sovereign debt and financial crises over the past two decades. Instead, operating with pre-existing regulatory gaps in financial markets and relying on private governance regimes designed and dominated by advanced economies to govern these new forms of development finance can reinforce existing economic and geopolitical asymmetries between countries.

Moreover, the enmeshment of public and private finance will insert countries into global financial markets and engagements with commercial investors in a way that will bring public institutions into sharp contact with regulatory regimes commonly associated with commercial investments, capital markets and corporate activity. Entry into new financial markets will inevitably have implications for developing countries’ broader international legal obligations in the longer term, concerns that are not ameliorated by current gaps in the architecture governing international financial markets and sovereign financing.

Instead, the privatisation of development finance is leading to greater privatisation of regulation where economic sectors, traditionally regulated by the state, will slowly be transferred to private governance regimes with knock-on impacts on civic accountability and transparency of public action. The ‘creation of new legal regimes and practices and the expansion and renovation of some older forms’ as a consequence of this shift to SDI markets can ‘have the effect of replacing public regulation and law with private mechanisms, sometimes bypassing national legal systems’.^133^ Further, as Picciotto has argued, the move towards ‘private institutional legal forms’ under this new re-engineered landscape of states and markets in public finance and elsewhere has been shown to be ‘ill-suited to managing the wider social responsibilities which decentred regulation requires them to accept’.^134^

Failure to account for these regulatory gaps will not only impact on the efficacy of SDI markets to deliver sustainable development objectives, but will paradoxically undermine efforts to do so, including by reinforcing pre-existing economic and geopolitical asymmetries between developing countries and industrialised economies and creating more platforms for wealth accumulation by northern-based private investors at the expense of global redistribution and collective financing of global public goods.

## Footnotes

1 Blended Finance Innovators (2016).

2 Bayliss and Romero (2021); Gabor (2021); UN Economic Commission for Africa (2020).

3 World Bank (2015).

4 Gabor (2021); Jafri (2019).

5 For ease of reference, the paper uses the term ‘sustainable development investing/investments’ (SDI) to refer to the diverse range of investment strategies and financial products that aim to raise capital and fund a similarly broad range of products, services, operations and programmes that meet global sustainable development objectives, including sectors mapped under the United Nations (UN) Sustainable Development Goals (SDGs), such as health, education, energy, poverty reduction, economic growth, gender equality and climate change mitigation and adaptation, to name a few (GISD, undated).

6 Kaul and Conceição (2006).

7 See Bayliss and Van Waeyenberge (2018), Gabor (2021) and Mawdsley (2018) for details on the longer historical relationship between international development and private sector engagement and promotion.

8 UN (2015), para. 49.

9 Tan (2021), p 17.

10 DFIs are bilateral or multilateral institutions controlled or owned by national governments that lend to or invest in private sector entities in developing countries. They are generally funded by governments through their ODA or other development aid budget and also raise capital through banks and financial markets. See, for instance, Romero (2014), p 7.

11 Romero (2014), p 7; IFC (2021). See also Sect. 2.2 for further discussion.

12 Bayliss and Romero (2021), p 6.

13 Jones (2021).

14 OECD and UNDP (2020), p 4.

15 Global Sustainable Investment Alliance (2018), p 8.

16 UN (2021), p 18; CFLI et al. (2020), p 13.

17 Gabor (2021), p 433.

18 See Babb and Kentikelenis (2021) for a discussion on the history of the Washington Consensus and the role played by IFIs, notably the International Monetary Fund (IMF) and the World Bank, in operationalising the policy prescriptions of this ‘ideational’ infrastructure.

19 Gabor (2021), p 441; Mawdsley (2018), p 192.

20 Bigger and Webber (2020), p 40.

21 Ibid.; Gabor (2021), p 443.

22 Tan (2021), pp 6–7.

23 See Pistor (2019), pp 2–4. Pistor’s landmark work on ‘the code of capital’ expands on previous scholarship exploring the operation of law and regulation in constituting markets for economic activity (see, e.g., De Sousa Santos (2002); Halliday and Carruthers (2009); Picciotto (2011)) by exposing how law encodes ‘assets’ – material or intellectual – as capital in order to render them, among other things, durable and convertible for the purpose of wealth generation for the asset owner.

24 See Gabor (2021), p 433; Pistor (2019), pp 2–4; Tan (2021), p 8.

25 Mawdsley (2018), p 192.

26 Ibid.; Bigger and Webber (2020), p 40.

27 Amacker and Donovan (2021), p 36; IPSF (2020), pp 13–20.

28 UN and UNEP (2019), pp 4–5, 16–17.

29 Asset classes are financial instruments with similar financial characteristics and sharing similar regulatory structures. Development of a new asset class includes ‘the adoption by the investment community of metrics, benchmarks and ratings that standardize performance and risk measurement’. See O’Donohoe et al. (2010), pp 8–9.

30 Boitreaud et al. (2020), p 31.

31 These frameworks include the development of voluntary guidelines for issuance of green, social and sustainability bonds, notably the Green Bond Principles (GBP), Social Bond Principles (SBPs) and Sustainability Bond Guidelines (SBGs). See ICMA (2020). See also the ICMA website https://www.icmagroup.org/sustainable-finance/ (accessed 9 May 2021).

32 IBRD (undated); IFC (2020a).

33 IBRD (2021), p 4.

34 IFC (2020b).

35 AIIB (2021); Bruni (2020).

36 World Bank (2018a).

37 AFD (2020); Davies et al. (2020).

38 Le Houérou (2018).

39 See OECD (2021), p 6. The OECD classifies ODA as resource transfers to developing countries and multilateral institutions from donor countries which are: (1) provided by official agencies, including state and local governments, or by their executive agencies; (2) concessional (i.e., grants and soft loans) and administered with the promotion of the economic development and welfare of developing countries as the main objective. Concessionality of loans is now calculated according to a new system of ‘grant equivalents’ introduced in 2019. Negotiations remain ongoing on the appropriate measurement of private sector instruments which include some of the forms of blended finance and derisking instruments used to catalyse SDI markets, but most private sector development activities by donor countries can be recorded as ODA if they meet the established criteria.

40 Caio (2018), p 3.

41 Park (2018a), p 254.

42 UN (2021), pp 59–71.

43 Amacker and Donovan (2021), p 19.

44 Ibid.; UN and UNEP (2019), p 10.

45 Environmental Finance (2020).

46 World Bank (2018b).

47 IFC (undated).

48 Ibid.

49 IFC (2021).

50 UNCTAD (2019a), p 75.

51 Alexander (2017); World Bank (2017), p 2.

52 UNDP (2021), p 3.

53 Craig and Porter (2006); Pahuja (2011); Tan (2011).

54 Ibid. See also Anghie (1999) and Swablowski (2007).

55 Karwowski (2019), p 1001.

56 Feigenbaum and Henig (1994), pp 185–186.

57 Karwowski (2019), p 1002.

58 Davis (2009), p 172.

59 Karwowski (2019), p 1014.

60 Karwowski (2020); Park (2018a); Park SK (2019); Roy et al. (2018); Tan (2019); Tan (2021).

61 Roy et al. (2018).

62 UNCTAD (2019a), pp 65–78.

63 Ibid., pp 86–87.

64 Use of proceeds and project evaluation and selection criteria are deployed by the issuer of labelled bonds to disclose where and how funds raised would be deployed, and they must conform with the overall criteria or taxonomies established by standard-setting bodies such as the ICMA, while reporting criteria require issuers to report regularly on the use of these funds. See Mathew (2018); Park (2018b), pp 12–14. A robust earmarking exercise would involve ‘integration of the solicitation of financing, the sale of the financial instrument, the selection of the project, and the allocation of funds into a sub-account or a sub-portfolio prior to disbursement’. See Park (2018b), p 12.

65 Park (2018b), p 17; see also Dadush (2015), pp 172–184; Gabor (2021), pp 12–18; Tan (2021), pp 12–15.

66 Soederberg (2007a, b).

67 Boitreaud et al. (2020), p 21.

68 Dadush (2012), p 394.

69 Ibid.; Dadush (2015); Tan (2021).

70 For examples of the extensive literature on this, see Broome and Quirk (2015); Davis et al. (2012). For a detailed discussion on their application in the SDI market and other forms of private finance for development, see Tan (2021).

71 Dadush (2012), p 425.

72 Ibid.

73 Park (2018b), p 26.

74 Boitreaud et al. (2020), p 18; ICMA (2018).

75 Ibid.

76 Petry et al. (2021), p 154.

77 See UN (2015), para. 127. See also UN (2015), para. 58; OECD (2005); OECD (2008). The AAAA specifically recognises ‘the importance of national ownership of the post-2015 development agenda’ and commits to aligning ‘activities with national priorities’ and ‘reducing fragmentation’ as well as promoting ‘country ownership’ in international development finance cooperation and policymaking.

78 Tan (2019), pp 311–322.

79 See, for example, Asian Development Bank (2018); IBRD and IDA (2015).

80 Spotlight on Corruption (undated).

81 Romero (2014); Vervynckt (2015).

82 UNCTAD (2019a), p 93.

83 IFC (2012), para. 11(a).

84 Ibid.

85 Publish What You Fund (2021), p 3.

86 Ibid., p 34.

87 Park (2018b), p 26.

88 Murray (2021).

89 Mathew (2018), pp 312–313.

90 Park (2018b), pp 30–32; Gabor (2021), pp 15–17.

91 Park (2018a), pp 250–251.

92 Roy et al. (2018).

93 Ibid.

94 Feigenbaum and Henig (1994), p 186.

95 Kovick (2018).

96 Ibid.; Park S (2019).

97 Bradlow (2010); Park S (2019).

98 See the GRS website: https://www.worldbank.org/en/projects-operations/products-and-services/grievance-redress-service; the Inspection Panel website: https://www.inspectionpanel.org/ and the CAO website: http://www.cao-ombudsman.org/ (all accessed 27 May 2021).

99 Shamir and Weiss (2012), p 129.

100 LeBaron et al. (2017).

101 De Lacey (2021).

102 UNCTAD (2019b), p 75.

103 Akyüz (2018), p 85.

104 UNCTAD (2019b), p 35.

105 Akyüz (2018); UN (2021), p 132.

106 OECD (2020).

107 Patricio Ferreira Lima (2020).

108 Ibid.

109 UNCTAD (2019b), pp 75–76.

110 Ibid., p 76.

111 Dillain (2020).

112 Dadush (2015), pp 191–192.

113 Park (2018a, b), p 6.

114 Dadush (2015), p 173; Park (2018a), pp 240–241.

115 Dadush (2015), pp 173–178.

116 Banahan (2019), pp 849–850; Rose (2019).

117 Boitreaud et al. (2020), p 21.

118 Banahan (2019), pp 849–850; Rose (2019), pp 75–76.

119 UNCTAD (2020), pp 114–115.

120 Ibid.; Akyüz (2018); Gelpern (2018).

121 UNCTAD (2020), p 113.

122 Ibid.; Akyüz (2018), pp 12–15; Gelpern (2018), pp 329–330.

123 Gelpern (2018).

124 Ibid.

125 Wheatley (2021).

126 IMF (2021).

127 Munevar (2021), p 4.

128 Ibid.

129 Ibid.

130 Institute of International Finance (2021).

131 Ibid.

132 Ibid.; see further Finance for Biodiversity Initiative (2021).

133 Sassen (1999), p 412.

134 Picciotto (2017), p 685.
